# Supplementary material for: Exploring supportive roles for global north investigators in global health research
Source: PLOS Glob Public Health. 2025 Apr 23;5(4):e0004358. doi: 10.1371/journal.pgph.0004358 (PMC12017523; doi:10.1371/journal.pgph.0004358)
Supplement: Attachment — Submitted filename: Response to the Review.docx [file pgph.0004358.s002.docx]

Response to the Review

Reviewer comments are in Italics and our point-by-point response is given in plain text font below. Significant changes are reflected in tracked changes in the main manuscript text.

*1. Provide a brief overview of the tenure process in institutions in the Global North*

We have now provided this in the second paragraph of the introduction of the revised manuscript.

*2. Expand on the conceptual framework in your Discussion section: How do partners from the Global South or pre-workshop participants conceptualize decolonization and anti-racist praxis?*

We agree that this is an important perspective to address. Because it is new information, we added this in a concluding paragraph in the results section. It seemed a good way to highlight voices from the Global South.

*3. Can you tell us more about the following points in the Discussion section: How are you involved in the decolonization movement? And how does your research contribute to the decolonization processes (in theory and practice) in the field of Global Health?*

We appreciate this feedback. We interpreted this feedback as a way to describe our co-author relation to the decolonization movement (similar to positionality statements that are increasingly included in methods sections of anthropological papers). As such, we placed a new section labeled 'Co-author engagement in the decolonization movement’ in the methods section.

*4. Could you elaborate on the group dynamics and discussions during the pre-workshop sessions? Were there any diverging perspectives, and what insights do these offer into the ongoing decolonization process?*

We agree that this is an important issue to address. We discuss this further in the limitations section of the paper.
